# Explaining the high PM_10_ concentrations observed in Polish urban areas

**DOI:** 10.1007/s11869-015-0358-z

**Published:** 2015-07-08

**Authors:** Magdalena Reizer, Katarzyna Juda-Rezler

**Affiliations:** Faculty of Environmental Engineering, Warsaw University of Technology, Nowowiejska 20, 00-653 Warsaw, Poland

**Keywords:** PM_10_ episode, Poland, Coal combustion, Source apportionment, PCA-MLRA, Backward trajectories

## Abstract

The main goal of this paper is to identify the drivers responsible for the high particulate matter concentrations observed in recent years in several urban areas in Poland. The problem was investigated using air quality and meteorological data from routine monitoring network, air mass back trajectories and multivariate statistical modelling. Air pollution in central and southern part of the country was analysed and compared with this in northern-eastern “The Green Lungs of Poland” region. The analysis showed that in all investigated locations, there is a clear annual cycle of observed concentrations, closely following temperature-heating cycles, with the highest concentrations noted in January. However, the main drivers differ along the country, being either connected with regional background pollution (in the central part of the country) or with local emission sources (in the southern part). The occurrence of high PM_10_ concentrations is most commonly associated with the influence of high-pressure systems that brought extremely cold and stable air masses form East or South of Europe. During analysed episodes, industrial point sources had the biggest (up to 70–80 %) share in PM_10_ levels on the days with maximum PM pollution, while remote and residential/traffic sources determined the air quality in the early stages of the episodes. Principal component analysis (PCA) shows that secondary inorganic aerosols account for long-range transported pollution, As, Cd, Pb and Zn for industrial point sources, while Cr and Cu for residential and traffic sources of PM_10_, respectively.

## Introduction

Particulate matter (PM) is a complex mixture of solid and liquid particles with varying physical, mineralogical and chemical characteristics dependent on categories identified according to their aerodynamic diameter, as either ultrafine PM_0.1_ (particles with an aerodynamic diameter, *d*_a_ < 100 nm), fine PM_2.5_ (*d*_a_ < 2.5 μm), coarse PM_10–2.5_ (*d*_a_ > 2.5 and *d*_a_ < 10 μm) or PM_10_ (*d*_a_ < 10 μm). Common chemical constituents of PM include crustal material, sea salt, organic carbon (OC), elemental carbon (EC), secondary inorganic aerosols (SIA including sulphates, nitrates, ammonia) and trace elements (TEs), as well as particle-bound water and unspecified compounds. Particles can either be directly emitted from natural sources and virtually all kinds of anthropogenic activities, or be formed in the atmosphere from other pollutants by gas-to-particle conversion processes. So far, numerous studies have shown the association of adverse health effects with exposure to PM over both short (days) and long (a year or more) periods, ranging from modest temporary changes, through increased risk of symptoms requiring hospital admission and increased risk of death from cardiovascular and respiratory diseases or lung cancer (e.g. Pope et al. 2002; Brunekreef and Forsberg 2005; Rückerl et al. 2011). Most recently, the specialized cancer agency of the World Health Organization (WHO), the International Agency for Research on Cancer (IARC) concluded that there is strong evidence that exposure to outdoor air pollution, and PM specifically, is associated with increases in genetic damage, including cytogenetic abnormalities, mutations in both somatic and germ cells and altered gene expression, which have been linked to increased cancer risk in humans and thus classified outdoor air pollution and particulate matter from this pollution as carcinogenic to humans—IARC group 1 (Loomis et al. 2013). According to the latest data, atmospheric PM pollution constitutes the 6th leading risk factor (among 43 ranked), which corresponds to over 3 million deaths worldwide every year (Lim et al. 2012). If no new policies are implemented, by 2050, outdoor air pollution is projected to become the top cause of environmentally related deaths worldwide (OECD 2012).

Since 1990, global emissions of primary PM and main precursors of secondary PM have either declined (PM_10_, SO_2_, NMVOCs) or slightly increased (NO_x_, NH_3_), but there is strong spatial variability, with Europe and North America continuing emission reduction and increasing role of Asia and Africa (based on data from the Emissions Database for Global Atmospheric Research—EDGAR v. 4.2). However, poor urban air quality due to high concentrations of PM remains a major public health problem worldwide. Severe PM episodes are occurring repeatedly in many cities all over the world, e.g. in Cracow and Warsaw (January 2006), New Delhi (November 2011), Athens (December 2012), Beijing (January 2013), Salt Lake City (January 2013), London (February 2014) and Paris (March 2014). According to the latest report of the European Environmental Agency, up to a third of the European Union’s (EU) urban population is exposed to levels of PM_10_ exceeding daily air quality limit value (50 μg m^−3^), while the exposure to PM_10_ levels that do not meet the annual air quality guideline (20 μg m^−3^) set by the WHO is significantly higher, comprising over 80 % of the EU urban population (EEA 2014). Moreover, only 2 % of the global urban population is living in areas where PM_10_ concentrations are lower than WHO air quality guideline (OECD 2012).

Therefore, it is crucial to gain knowledge about the types of emission sources responsible for severe PM events. To address this issue, different source apportionment (SA) methods are widely used in worldwide studies. Two main types of approaches are employed: (1) bottom-up methods based on numerical dispersion modelling and (2) top-down methods applying receptor modelling (RM) techniques. The usage of dispersion models, which simulate aerosol emission, formation, transport and deposition, basing on detailed emission inventories as well as meteorological and topographical data, is limited in SA analyses by the accuracy of the input data, especially emission data (Juda-Rezler 2010; Kiesewetter et al. 2015). However, these models are frequently preferred when natural sources of PM are of special interest (Fragkou et al. 2012). The most regularly used bottom-up models are the Eulerian chemical transport models, followed by the Lagrangian ones. On the contrary, RM models based on multidimensional statistical analysis of ambient PM concentrations and its chemical composition are independent from emission inventories and meteorological data. The number of RM tools, ranging from simple techniques applying elementary mathematical calculations and basic physical assumptions, up to complex models requiring pre- and post-processing of data, are currently available. Until 2005, principal component analysis (PCA) was the most frequently used RM model (Viana et al. 2008), while in the recent years, shifting towards more advanced methods, which require also more input data—such as positive matrix factorization (PMF) and chemical mass balance (CMB)—is observed (Belis et al. 2013). PMF method requires uncertainties of ambient concentrations, while CMB requires emission profiles of relevant sources as well as uncertainties of both ambient concentrations and emission profiles. Meta-analysis of 108 European SA studies conducted by Belis et al. (2013) defined six major source categories for PM in Europe: atmospheric formation of secondary inorganic aerosol, traffic, resuspension of crustal/mineral dust, biomass burning, (industrial) point sources and sea/road salt. A review of SA techniques conducted by Johnson et al. (2011) identified 11 common PM source categories in 18 developing countries of Asia, Africa and Latin America, grouped into 4 main types: (1) dust emissions, including road dust, soil dust, resuspension, fugitive dust and construction; (2) transport (gasoline, diesel); (3) industrial activities, including coal and oil burning, brick kilns and power plants; as well as (4) non-urban, including biomass burning, long-range transport and marine sources.

In Poland, as in the majority of central-eastern European countries, the dependence of economy on coal is still higher than in western European countries. Hard coal and lignite amount to approximately 50, 80 and 77 % in the structures of primary energy, electricity and heat consumption, respectively. It is well recognized that coal combustion is one of the main source of primary PM and precursor gases emissions. However, according to our knowledge, there has no previously been a study differentiating the source profile of coal combustion in different utilities, i.e. domestic (residential) stoves/boilers and industrial high-efficiency boilers.

Therefore, the main goal of this paper is to distinguish different types of PM emission sources as well as to discern coal combustion in industrial and domestic sources that determine high PM_10_ concentrations recorded in Polish urban areas. In recent years, a number of PM episodes varied in spatial range and strength, which occurred in Polish cities (Reizer 2013). In general, they are occurring in different synoptic situations; however, the largest frequency is observed in anti-cyclonic conditions with the air masses coming from eastern, south-eastern and southern directions (Reizer 2013; GIOŚ 2013). For the present study, two such wintertime episodes of January 2009 and January 2010, connected with eastern and southern inflow of air masses, have been selected for investigation.

## Materials and methods

### Selection of episodes

In general, there is no definite rule for selecting episode value from a measurement database and three different approaches are usually applied. The most commonly used method is to assume the daily air quality limit value for a selected pollutant as a threshold value (e.g. Kukkonen et al. 2005; Muir et al. 2006; Aarnio et al. 2008; Im et al. 2010). In some studies, either the maximum value is classified as an episode (e.g. Bessagnet et al. 2005; Amodio et al. 2008) or a given threshold based on personal experience is assumed (e.g. Niemi et al. 2009). Finally, the concentration exceeding the 75th percentile (e.g. Karaca et al. 2009) or the 95th percentile (e.g. Chu 2004) is marked as an episode. In this study, the severe episode is defined as a situation with the daily PM_10_ concentration at the urban background site exceeding 100 μg m^−3^ (200 % of the EU limit value) for at least three subsequent days. According to the above definition, 77 PM_10_ episodes that occurred in analysed cities in 2005–2012 period were recognized. Two wintertime episodes of 7–16th January 2009 and 22–28th January 2010, connected with eastern and southern inflow of air masses, were investigated and hereinafter are referred to as episodes 1 and 2, respectively.

### Characteristics of investigated cities

**Fig. 1:**
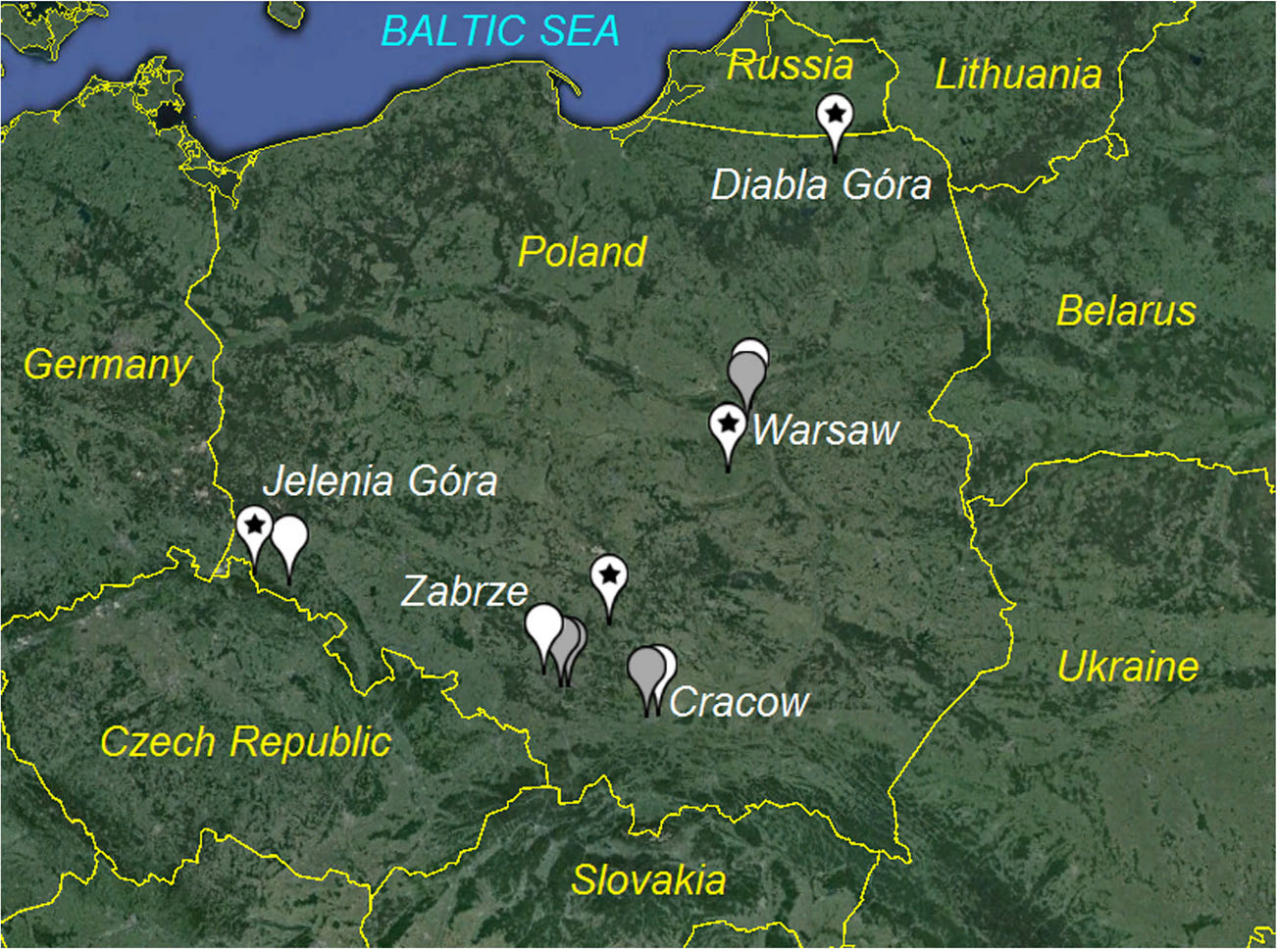
Location of urban background (*white*), traffic (*grey*) and regional/rural background (*marked by stars*) air quality monitoring sites

The air quality during the episodes was considered in four Polish cities, situated in central (Warsaw) and southern (Cracow, Zabrze, Jelenia Góra) part of the country (Fig. 1).

The selected cities are characterized by different conditions with respect to climate, topography and emission sources.

Warsaw, the capital city of Poland, is located in the Mazovian Lowland in central Poland. With 1.7 million inhabitants, it is the largest agglomeration in the country and 11th largest city in the EU-28 (EUROSTAT 2013). Nonetheless, it is characterized by relatively good air quality regarding the main pollutants. The majority of the city’s households are connected to a central heating supply system which greatly limits the pollution that originates from private use of fossil fuels for domestic heating. The most important PM local emission sources remain road transport and cogeneration plants (AQP for Warsaw 2013). Due to the lack of a real bypass road, Warsaw is one of the most congested cities in Poland, where most of the traffic is routed through the city streets, which are quite narrow in many areas.

Cracow, with a population of approximately 760,000 inhabitants, is the second largest city in Poland, situated in the Lesser Poland close to the most extensively industrialized and highly polluted Upper Silesian region. The city is also influenced by surrounding industrial sources that include steelworks (the second largest steel plant in Poland) as well as coking, electric power and cogeneration plants. The most important local emission sources are residential burning of coal/wood and road transport (AQP for Lesser Poland Voivodeship 2013). Moreover, Cracow, located in the wide valley of the Vistula river, is enclosed by shallow hills that aggravates the dispersion of pollutants.

The city of Zabrze together with other ten cities belongs to the Silesian Metropolis which population counts over 2.7 million inhabitants. It is located in the Upper Silesia region of Southern Poland, where coal mining and metallurgy, as well as the coke factories, are the major industrial activities. The largest steel plant in Poland is also located in the neighbourhood of the city. However, the most important local emission source in Zabrze is burning of coal/wood in small residential boilers that are used in large part of the city (AQP for Silesian Voivodeship 2010).

Jelenia Góra which constitutes a health resort area is situated in the Lower Silesian region of Southern Poland, in the area belonging to the former “Black Triangle” extremely polluted region of Europe. The most important local emission source of PM is again the residential sector (AQP for Lower Silesian Voivodeship 2010). Air pollution dispersion in the city is aggravated due to its location in the valley surrounded by the mountains on all sides, with the highest range—Karkonosze—in the south.

### Air quality monitoring sites

For each city, air quality monitoring data from urban background (UB), traffic (TR) and the nearest regional background (RB) sites were considered. Due to its character, rural background site in Diabla Góra (PL0005R) belonging to the European Monitoring and Evaluation Programme (EMEP) network was chosen as representative for continental background (Fig. 1). The nearest town (>10,000 inhabitants) and road (>50 cars per day) are situated at 20 and 16 km from this site, respectively. The characteristics of investigated air quality monitoring sites are given in Table 1.

**Table 1:** Characteristics of air quality monitoring sites

City-site name	Code	Label	Method of PM_10_ analysis	Longitude (° ′ ′′ E)	Latitude (° ′ ′′ N)	Altitude (m a.s.l)
Warszawa-Targówek	PL0143A	W-UB	(2)	21° 02′ 33′′	52° 17′ 36′′	85
Warszawa-Komunikacyjna	PL0140A	W-TR	(2)	21° 00′ 17′′	52° 13′ 00′′	103
Belsk IGPAN	PL0014A	W-RB	(2)	20° 47′ 30′′	51° 50′ 00′′	176
Kraków-Nowa Huta	PL0039A	C-UB	(3)	20° 03′ 07′′	50° 04′ 00′′	195
Kraków-Krasińskiego	PL0012A	C-TR	(3)	19° 55′ 20′′	50° 03′ 00′′	175
Zabrze-Skłodowskiej	PL0242A	Z-UB	(1)	18° 46′ 19′′	50° 19′ 12′′	255
Chorzów	PL0235A	Z-TR	(2)	18° 56′ 13′′	50° 15′ 00′′	285
Złoty Potok	PL0243A	Z-RB (C-RB)	(3)	19° 27′ 36′′	50° 42′ 36′′	291
Jelenia Góra-Cieplice	PL0189A	J-UB	(3)	15° 44′ 06′′	50° 51′ 36′′	341
Czerniawa	PL0028A	J-RB	(3)	15° 18′ 51′′	50° 54′ 36′′	645
Diabla Góra	PL0005R	–	(1)	22° 04′ 01′′	54° 08′ 00′′	157

(1) A reference gravimetric sampler (Standard EN 12341); (2) Tapered Element Oscillating Microbalance (TEOM®); (3) beta ray attenuation method (MP101M)

The data of PM_10_ mass concentrations for all sites and the concentrations of PM_10_ and its constituents for Diabla Góra site: TEs (As, Cd, Cr, Cu, Ni, Pb, Zn) and SIA (SO_4_^2−^, NH_3_ + NH_4_^+^, HNO_3_ + NO_3_^−^) were extracted from the Voivodeship air pollution networks which belong to the Polish Voivodeship Environment Protection Inspectorates. Data was also extracted from EMEP (http://www.emep.int) and Air-Base, European Air quality (http://acm.eionet.europa.eu/databases/airbase) databases. The data series fulfilling the criterion of 75 % completeness of the data sets within analysed periods were retained for the analysis. Depending on the monitoring site, concentrations of PM_10_ were determined by either (1) a reference gravimetric method (Standard EN 12341), (2) Tapered Element Oscillating Microbalance (TEOM®) or (3) beta ray attenuation method (MP101M) (Table 1). None of the two latter methods of PM_10_ measurements are considered to be the reference methods regarding the standard EN 12341, hence Poland, as the EU member state, is required to demonstrate that their methods yield results equivalent to the reference (EC 2010). However, up to now, the procedures for equivalency demonstration have not yet been accustomed. Therefore, herein, we have used the official data as they were reported to the EMEP and AirBase repositories. The analysis of TEs content was performed using inductively coupled plasma–atomic emission spectroscopy (ICP–AES), while sulphate and sum of nitric acid and nitrate, as well as sum of ammonia and ammonium were determined by capillary electrophoresis (CE) and spectrophotometry, respectively.

### Methodology

In order to identify the emission sources determining PM_10_ concentrations during the most severe episode noted in the history of air quality measurements in Poland (January 2006), the original method of PM source identification was applied in the study of Juda-Rezler et al. (2011). In the present paper, this method was further developed and extended. The influence of specific anthropogenic sources on PM_10_ levels during the episodes was investigated by the combination of different apportionment techniques applied to routinely available air quality and meteorological monitoring data, i.e. (1) analysis of the PM_10_ patterns at both urban and regional background monitoring sites, following the Lenschow approach (Lenschow et al. 2001); (2) analysis of the synoptic situation and variability of the local meteorological parameters; (3) analysis of the air mass back trajectories; and (4) receptor modelling using principal component analysis with multivariate linear regression analysis (PCA-MLRA).

The Hybrid Single-Particle Lagrangian Integrated Trajectory (HYSPLIT) model of the Air Resources Laboratory (ARL) of the National Oceanic and Atmospheric Administration (NOAA) was used, in order to calculate transport of air masses to each city (Draxler and Rolph 2013). The model was forced by the global reanalysis meteorological data produced by the National Weather Service’s National Service for Environmental Prediction (NCEP) to compute advection and dispersion of air parcels. Three-day backward trajectories arriving at the studied locations at 50, 100, 200, 500 and 700 m were generated. In Poland, winter is characterised by long-lasting ground-based inversions (also during daytime) and a weak convective mixing mainly around the midday (GIOŚ 2013; Godłowska et al. 2015). Such stagnant conditions were present also during elaborated episodes. Therefore, it was supposed that near-surface air quality (measured at receptor site) could be affected by sources along trajectories only when backward trajectory starts within mixing layer or ground inversion layer. Hence, the heights of trajectories starting points were situated in the bottom part of PBL.

In this study, the air pollution concentrations were obtained from routine air quality monitoring networks, which do not provide the data uncertainties. Thus, the use of more advanced receptor models such as PMF or CMB was precluded. Therefore, receptor modelling technique based on PCA with Varimax rotation was applied to TEs and SIA concentrations in order to identify the main sources determining the aerosol composition at the rural background site, while the Lenschow approach was employed to ascertain PM sources in urban areas. In order to facilitate comparison between the different seasons (spring, summer, autumn, winter), PCA was performed for each period separately, resulting in input data matrices of sizes between 10 × 70 and 10 × 90. The decision of selecting the number of principal components (PC) was based on the eigenvalue of the factors. Following the Kaiser criterion, only factors having an eigenvalue greater than 1.0 were retained for further analysis. The interpretation of the principal components was based on the variables with factor loadings with absolute values greater than 0.6. The contribution of each source to the PM burden was further quantitatively assessed by the means of MLRA procedure proposed by Thurston and Spengler (1985).

The statistical significance of the differences between mean PM_10_ concentrations in heating and non-heating season was assessed using Student’s *t* test. All statistical analyses were performed with STATISTICA software.

## Results and discussion

### PM_10_ levels

#### Seasonal variation

**Fig. 2:**
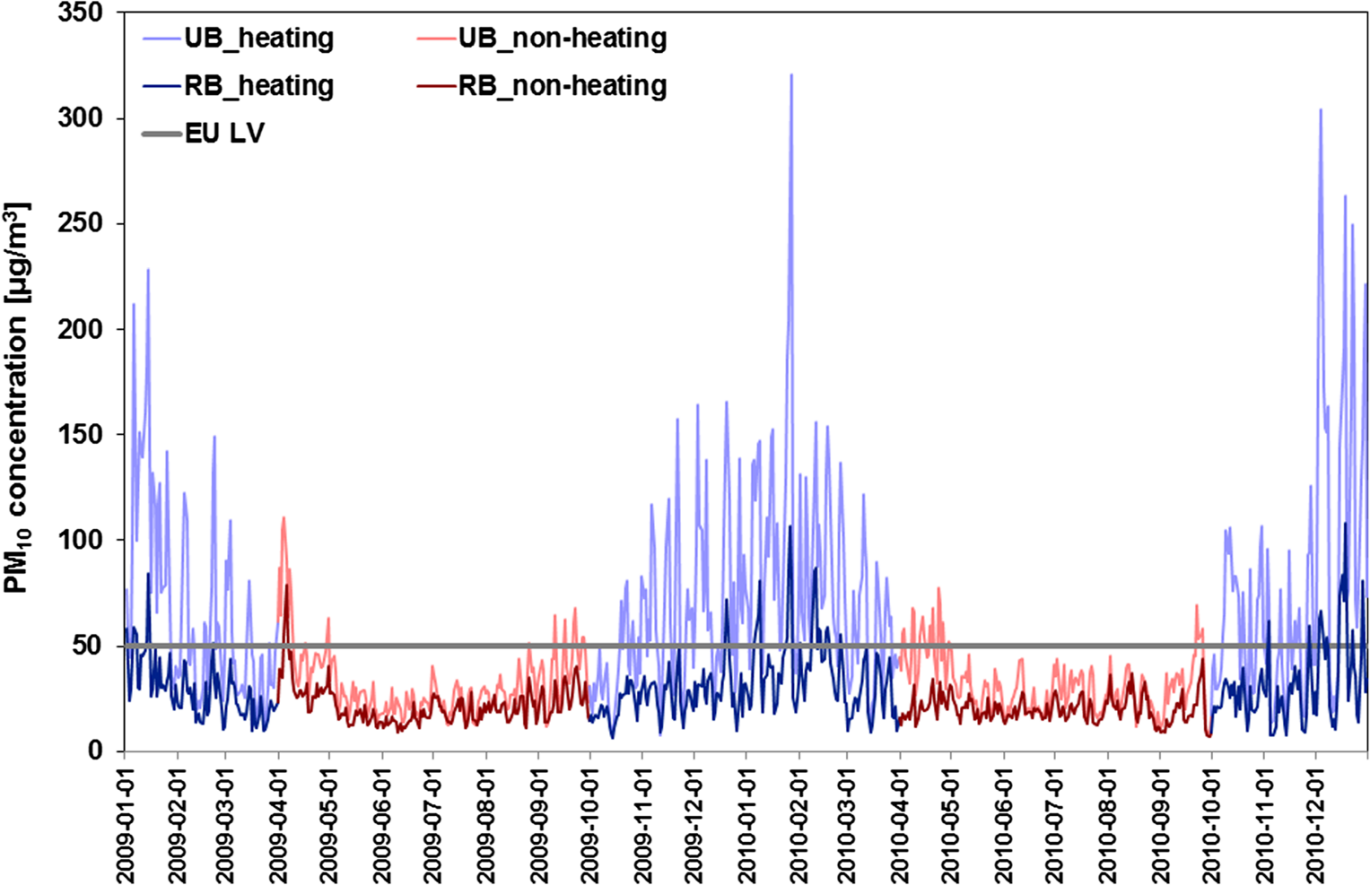
Patterns of daily PM_10_ concentrations (μg m^−3^) during the 2009–2010 period averaged for urban (*light lines*) and regional (*dark lines*) background monitoring sites. *Blue* and *red lines* indicate heating (October–March) and non-heating (April–September) seasons, respectively, while *grey solid line*—EU daily limit value for PM_10_ (50 μg m^−3^)

The seasonal variation of PM_10_ concentrations for the years 2009–2010 presents a common pattern for both urban and regional background sites (Fig. 2), which is defined by emission cycles of primary PM and precursors of secondary PM. The seasonal trend is characterized by two annual maxima: January–March and October–December, which corresponds with heating season in Poland. Student’s *t* test revealed that statistically significant differences between average urban background PM_10_ concentrations recorded during heating (65.0 and 83.6 μg m^−3^ in 2009 and 2010, respectively) and non-heating (32.4 and 31.9 μg m^−3^ in 2009 and 2010, respectively) season can be observed (*p* < 0.00001). The seasonal differences of the average PM_10_ concentrations for regional background sites for both analysed years are also statistically significant (*p* < 0.00001). In addition, the average PM_10_ concentrations for the urban background sites are significantly higher than for the regional ones (*p* < 0.00001). This could be explained as due to the larger PM emissions of urban origin.

#### Characteristics of air pollution episodes

**Fig. 3:**
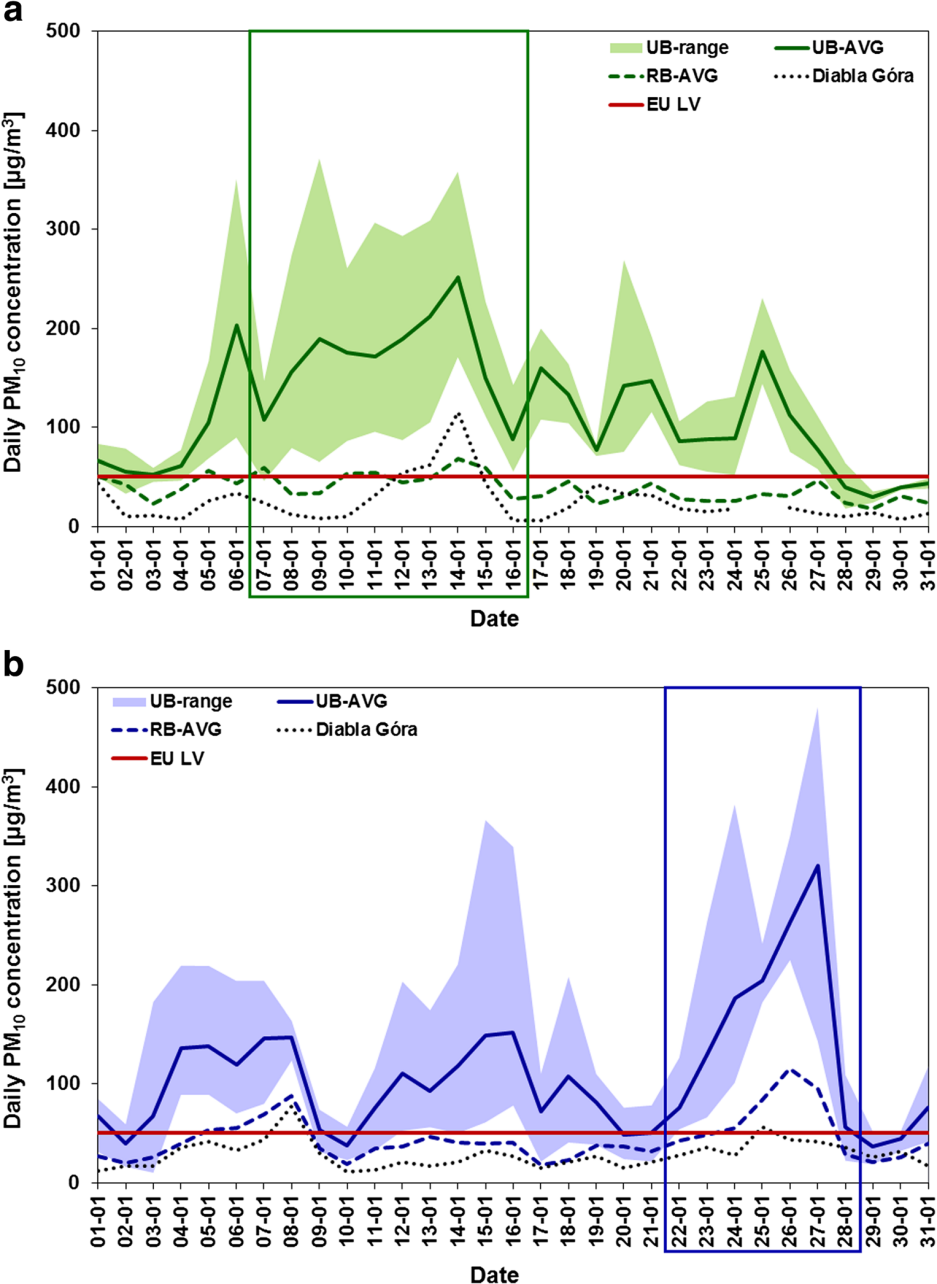
Patterns of daily PM_10_ concentrations (μg m^−3^) during **a** January 2009 (*green*) and **b** January 2010 (*blue*) averaged for urban (*solid lines*) and regional (*dashed lines*) background monitoring sites and at rural background EMEP site in Diabla Góra (*black dashed lines*). *Colour light backgrounds* indicate the range of PM_10_ concentrations at urban background sites, *colour frames*—the period of PM episodes, while *red solid lines*—EU daily limit value for PM_10_ (50 μg m^−3^)

Daily PM_10_ concentrations observed in Januaries 2009 (Fig. 3a) and 2010 (Fig. 3b) at both urban and regional background air quality monitoring sites indicate substantially increased pollutant concentrations during all investigated PM_10_ events being more pronounced in UB sites than in RB ones. Maximum PM_10_ levels in episode periods were found to exceed the daily EU air quality limit value for PM_10_ (50 μg m^−3^) from sevenfold in January 2009 (372 μg m^−3^) up to tenfold in January 2010 (481 μg m^−3^).

Episode 1 of 7–16th January 2009 occurred only in southern cities and can be regarded as the regional one. In northern and central Poland, the daily PM_10_ limit value was in general not exceeded. In Warsaw, the maximum daily PM_10_ concentration (160 μg m^−3^) was recorded in the same date as for the rest of cities (14th January); however, the exceedance of the limit value persisted shorter than 3 days and therefore PM_10_ pollution recorded in this city cannot be classified as an episode according to the definition set in the present study. In Zabrze and Jelenia Góra, the episode was characterized by two peaks of PM_10_ concentrations with the first peak being slightly higher (Fig. 3a). The maximum PM_10_ levels, up to 372 and 181 μg m^−3^ in Jelenia Góra and Zabrze, respectively, were observed on 10th and 14th January. In Cracow, three maxima of PM_10_ levels on 7th, 9th and 14th January were recorded with the third peak being the highest (225 μg m^−3^). During the episode, the patterns of daily PM_10_ concentrations at RB sites were analogous to those registered at UB sites with much lower values up to 25 and 90 μg m^−3^ at J-RB and Z-RB sites, respectively. At the rural background site in Diabla Góra, the maximum PM_10_ level was recorded on 14th January (115 μg m^−3^). It was the highest PM_10_ concentration recorded at this site during the period of 2005–2012 (Reizer 2013).

Episode 2 of 22–28th January 2010 was preceded by two episodes with lower PM concentrations: the 2–10th and the 11–20th January (Fig. 3b). During episode period, high PM_10_ levels were observed at monitoring sites across the whole country. In three out of four cities, the episode was characterized by only one peak of high PM_10_ concentrations observed on 27th January. At rural background site in Diabla Góra, one peak concentration (56 μg m^−3^) was also recorded; however, it occurred 2 days before the maximum PM_10_ levels registered at the rest of the sites. Two peaks of PM_10_ levels were observed on 24th and 27th January with the highest value of 480 μg m^−3^ in Jelenia Góra. During the episode, the patterns of daily PM_10_ concentrations at RB sites were similar to those recorded at UB ones with the maximum values up to 70 μg m^−3^ at J-RB site and around 140 μg m^−3^ at the rest of RB sites.

The analysis of daily PM_10_ concentration patterns at UB and RB sites indicates that during the episodes, urban sources were significantly involved in the build-up of pollution events in all cities. However, the regional background was also quite high with the ratios of RB/UB increasing from the southern (densely industrialized) to the central part of Poland (Table 2). The highest contribution of regional pollution was found in both Warsaw and Zabrze, amounting to more than a half of the urban episodes (RB/UB = 0.57 and 0.54–0.55, respectively). In contrast, for the southern city of Jelenia Góra, the regional background pollution was the lowest (RB/UB = 0.06–0.15), indicating that during all the episodes air quality in this city was strongly determined by the local “hot-spot” emission sources. At the same time, comparably high TR/UB ratios were obtained for Cracow during episodes 1 (1.26) and 2 (1.21), indicating typical for urban areas traffic component to the overall urban pollution.

**Table 2:** Average (avg) PM_10_ concentrations measured during analysed PM episodes at urban (UB) or regional (RB) background and traffic (TR) monitoring sites

Episode	City	UB avg (μg m^−3^)	TR avg (μg m^−3^)	RB avg (μg m^−3^)	RB/UB	TR/UB
Episode 1	Cracow	132.1	165.9	64.1	0.49	1.26
	Zabrze	115.9	95.3	64.1	0.55	0.82
	Jelenia Góra	259.1	–	16.3	0.06	–
Episode 2	Warsaw	134.6	95.7	76.9	0.57	0.71
	Cracow	162.7	197.0	75.9	0.47	1.21
	Zabrze	141.6	117.2	75.9	0.54	0.83
	Jelenia Góra	266.7	–	39.6	0.15	–

### Synoptic situation and local meteorological conditions

**Fig. 4:**
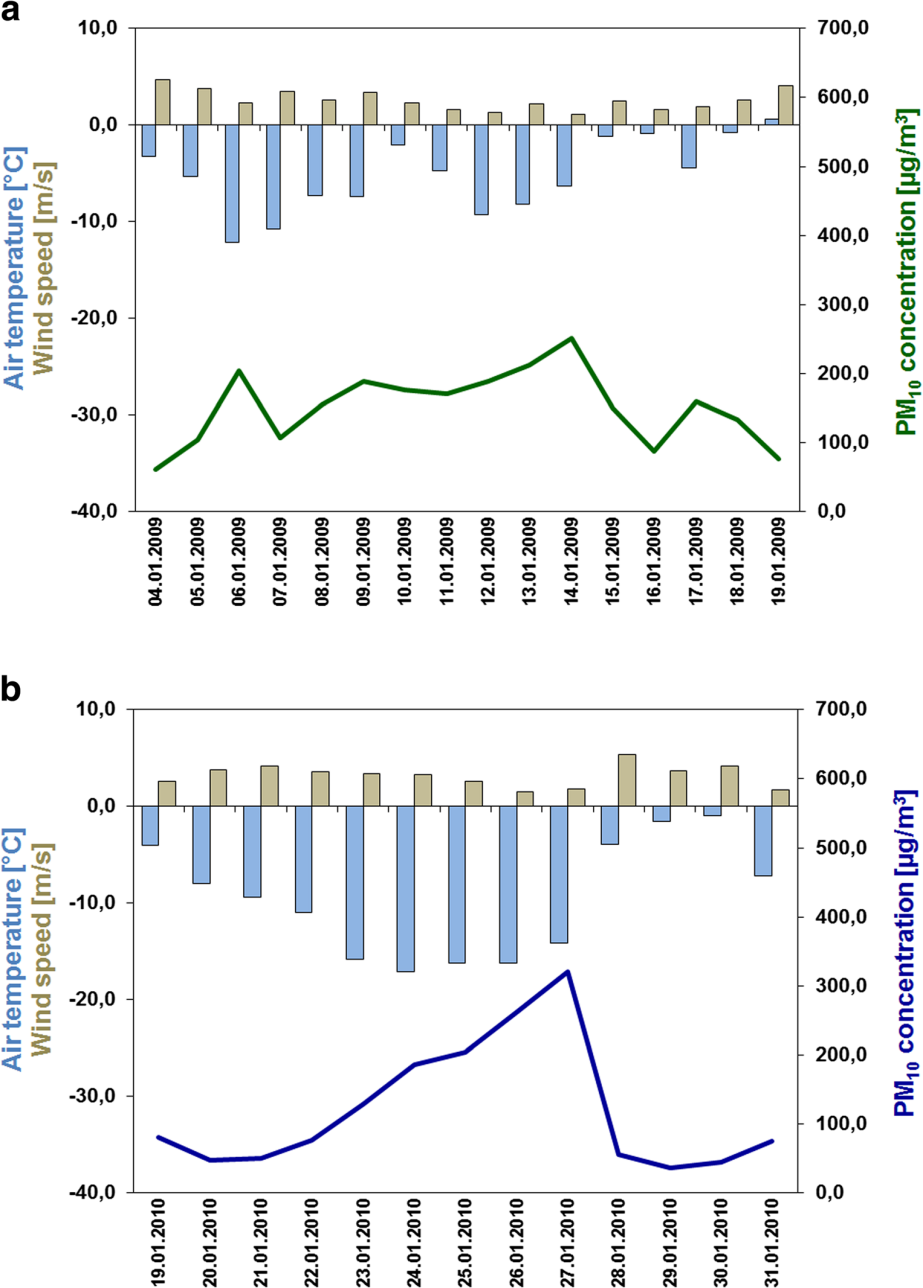
Patterns of daily PM_10_ concentrations (μg m^−3^) (*colour solid lines*) during the period of **a** 4–19th January 2009 and **b** 19–31th January 2010 averaged for urban background monitoring sites in comparison with daily mean of meteorological parameters: air temperature (°C) (*blue bars*) and wind speed (m s^−1^) (*beige bars*)

The patterns of the measured PM_10_ concentrations during the episodes can be explained by the variation of the local meteorological parameters (Fig. 4). During episode 1, southern part of Poland was under the influence of very stable high-pressure system with its centre over the Czech Republic in the early stage of the episode and over Hungary in the rest of the episode period, while the rest of the country was under the influence of other high-pressure systems. The maximum values of air pressure were observed on 11th January reaching 1030–1035 hPa. At the beginning of the episode (6–9th January), average daily temperature varied from −10 to −15 °C, falling down below −20 °C during night. Second, a smaller decrease of air temperature was noted on 12–14th January with minimum daily values close to −10 °C. Low wind speeds up to 5 m s^−1^, and below 2 m s^−1^ in the days with the highest PM_10_ concentrations were also recorded. During this episode, the peak PM_10_ concentrations occurred 2 and 3 days after the maximum air pressure and minimum air temperature in Zabrze and in both Cracow and Jelenia Góra, respectively.

During episode 2, Poland was under the influence of the Siberian High ridge with maximum value of 1040 hPa. Low air temperatures were observed with daily minimum below −17 °C in the southern cities (on 24th January) and −19 °C in the central city of Warsaw (on 25th January). Weak wind up to 5 m s^−1^ was also recorded, whereas in Zabrze, the wind speed decreased below 1 m s^−1^ during the days with the highest PM_10_ concentrations. Episode 2 was characterized by the occurrence of the peak PM_10_ concentrations up to 3 days after the maximum air pressure and minimum air temperature.

**Fig. 5:**
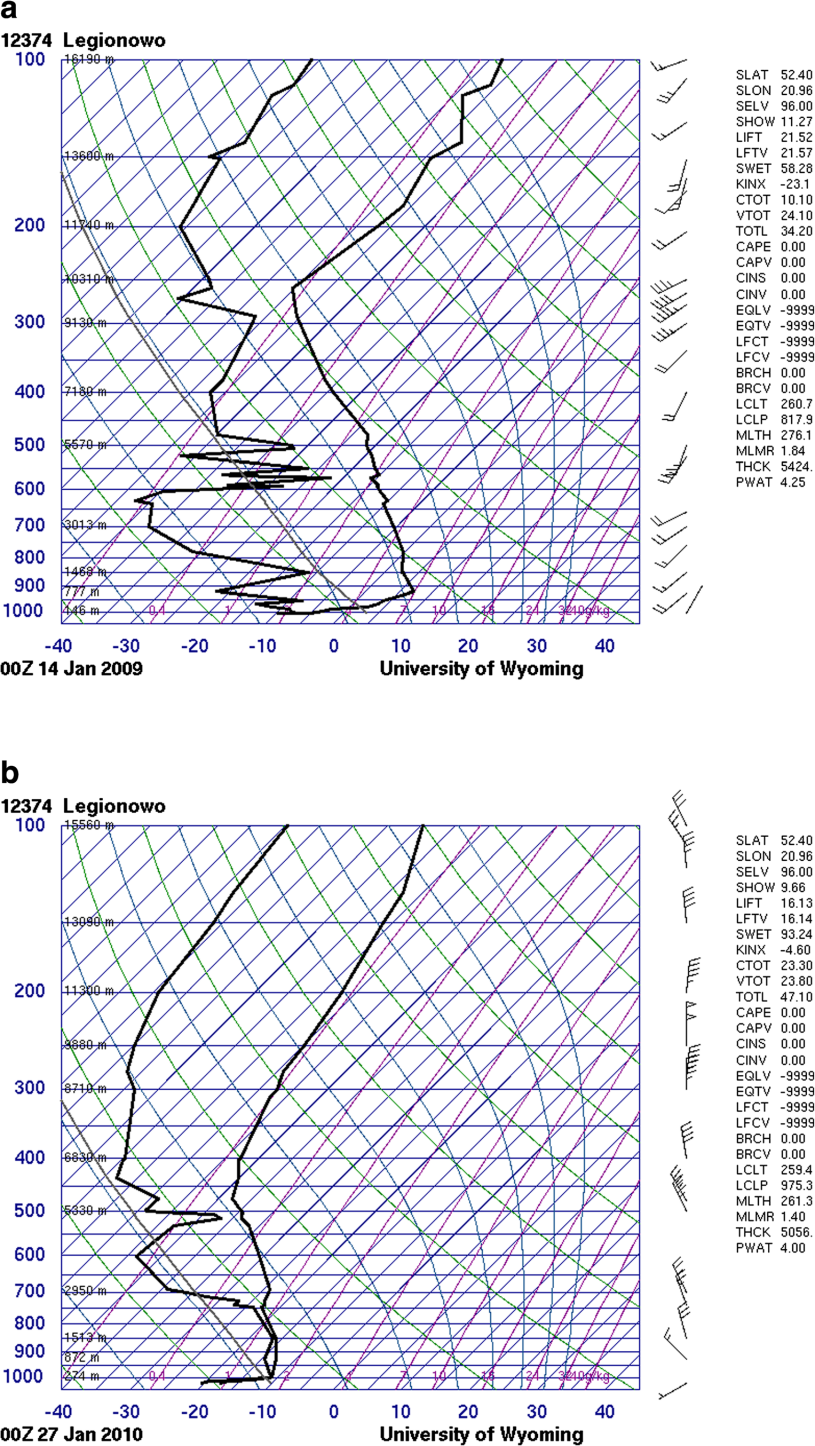
Vertical soundings from Legionowo station (central Poland) measured on **a** 14th January 2009 at 00Z and **b** 27th January 2010 at 00Z (http://weather.uwyo.edu/upperair/sounding.html)

The vertical soundings (skewT-logP diagrams), from the atmospheric sounding taken at Legionowo station in central Poland at 00Z on days with maximum PM_10_ concentrations during each episode, show that there were strong thermal inversions below 900 hPa (Fig. 5). For those days, the inversion layers were found to be approximately 700 m deep during both episodes. In the early stage of the episodes, the subsidence inversions were noted, followed then by the strong radiation inversions. The inversion layers together with high air pressure, extremely low air temperature and low wind speed impeded air pollution dispersion during episodes leading to the accumulation of air pollutants and fostering the formation of severe PM_10_ events.

### Air mass back trajectories

**Fig. 6:**
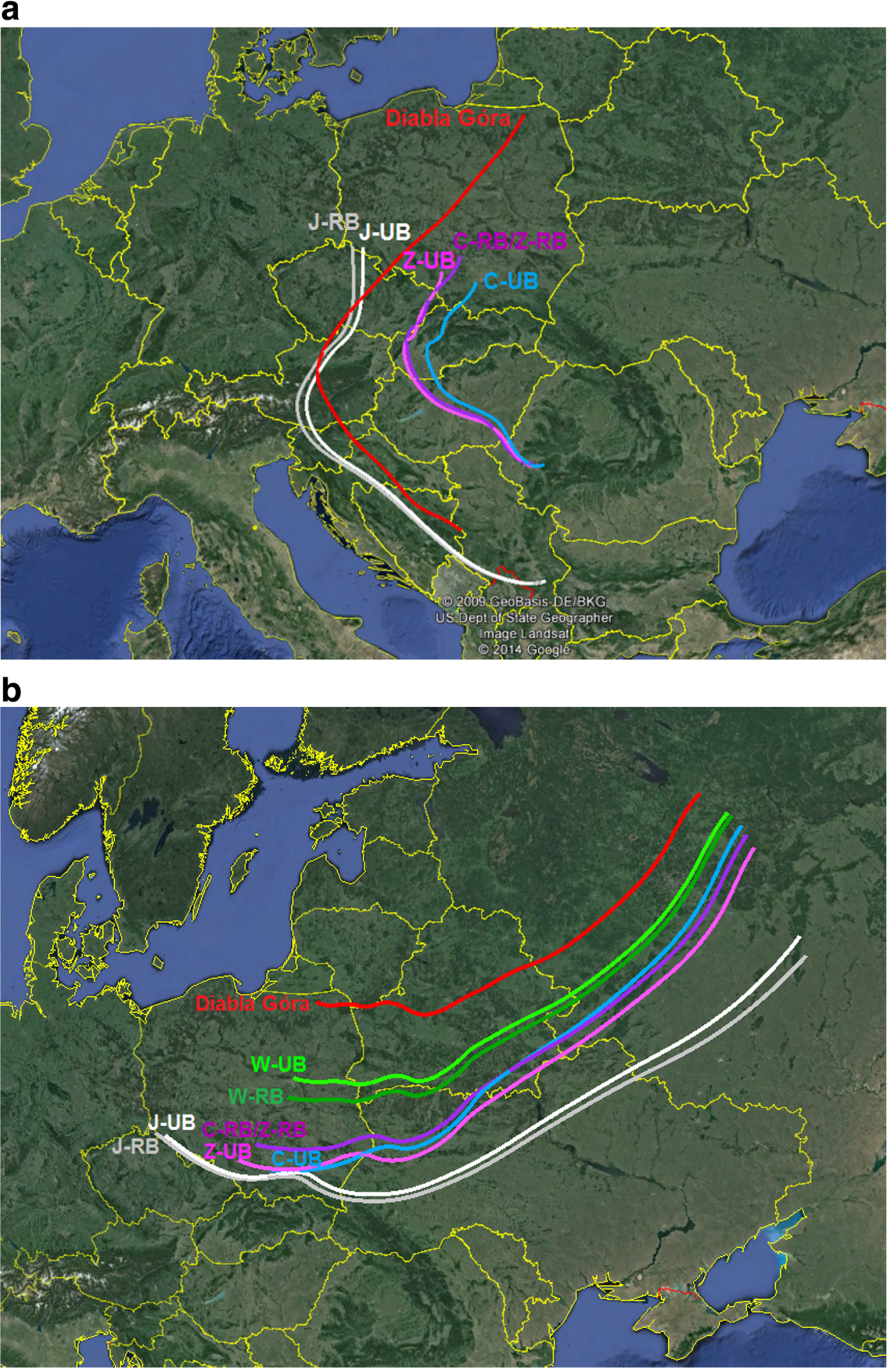
Three-day air mass back trajectories at the altitude of 50 m a.g.l. at urban (*light colours*) and regional (*shaded colours*) background monitoring sites of the four Polish cities: Warsaw (*green*), Cracow (*blue*), Zabrze (*purple*), Jelenia Góra (*white*) and at rural background EMEP site in Diabla Góra (*red*), calculated by HYSPLIT model at 00 UTC on **a** 14th January 2009 and **b** 25th January 2010

Transport of air masses to pairs of UB and RB sites of each city was examined by means of atmospheric 3-days back trajectories. Due to the fact that the air mass trajectories calculated for each day of the episode demonstrate similar pattern and that the trajectories determined at all altitudes were almost identical for each city, only back trajectories calculated at 50 m of altitude are presented in Fig. 6.

Analysis of air mass back trajectories shows that during the analysed episodes, all pairs of UB and RB sites were under the influence of the same air masses moving along the high-pressure system edges. During episode 1, the air masses arriving to Jelenia Góra and Diabla Góra were coming from Serbia and passing through Bosnia and Herzegovina, Croatia, Slovenia and Czech Republic and then from southern to northern Poland in case of Diabla Góra. On the contrary, the air masses arriving to Zabrze and Cracow were transported from the western part of Romania through Hungary and Slovakia. During episode 2, the air masses, coming mainly from the European part of Russia, were transported through Ukraine, Belarus and further over Poland.

The analysis of air mass back trajectories indicates that during episode 2, both UB and RB sites were under the influence of the same air masses of north-eastern origin that brought polar air masses from a Siberian high ridges coming mainly from central parts of European Russia and transported through Belarus and Ukraine. Thus, all investigated Polish cities appear to be under the influence of the same long-range transported air pollutants from remote sources in Eastern European countries. On the contrary, during episode 1, all types of sites were under the influence of a high-pressure system bringing air masses from Southern European countries to both types of sites. Furthermore, regional transport of air pollutants determining high PM_10_ concentrations during episodes is associated with the transport of air masses over Poland. The air masses arriving to the so-called “Green Lungs of Poland”, delimited in the north-eastern, highly forested and most sparsely populated region of the country, where the rural background site in Diabla Góra is located, brings there pollutants from both remote and regional industrial sources.

The results of air mass back trajectories analysis were confirmed by the air pollution roses calculated for Diabla Góra site (not shown here) which indicate that the highest concentrations of TEs occur at the southern or eastern direction of wind.

### PCA-MLRA analysis

PCA with Varimax rotation applied to TEs and SIA concentrations measured at Diabla Góra site allows to extract three principal components (PC) explaining up to 88 and 81 % of the total variance for the winters (January–March) of 2009 and 2010, respectively (Table 3). A major group of TEs that includes As, Cd, Pb and Zn was collocated in the PC1 which explain around 40 % of the total variance for both 2009 and 2010. The second set (PC2 with explained variance between 25 and 30 %) is characterized by the group of SIA components, however, lacking sum of ammonia and ammonium in winter 2009 and sulphate in winter 2010. The third group (PC3 with almost 16 % of explained variance) is represented mostly by Cr and Cu.

**Table 3:** Factor loadings determined in the PCA analysis with Varimax rotation of trace elements and SIA measured at rural background EMEP site in Diabla Góra during January–March 2009 and January–March 2010

PM_10_ component	January–March 2009			January–March 2010		
	PC1	PC2	PC3	PC1	PC2	PC3
As (ng m^−3^)	*0.92*	0.24	0.21	*0.82*	0.37	0.05
Cd (ng m^−3^)	*0.93*	0.17	0.12	*0.86*	0.09	0.37
Cr (ng m^−3^)	0.30	0.05	*0.92*	0.44	0.27	*0.62*
Cu (ng m^−3^)	0.04	*0.72*	*0.63*	0.30	0.30	*0.75*
Ni (ng m^−3^)	*0.65*	0.63	0.13	0.53	*0.68*	0.09
Pb (ng m^−3^)	*0.84*	0.41	0.26	*0.91*	0.28	0.21
Zn (ng m^−3^)	*0.71*	0.53	0.32	*0.91*	0.20	0.27
SO_4_^2−^ (μg(S) m^−3^)	0.53	*0.73*	0.13	0.49	0.32	0.52
NH_3_ + NH_4_^+^ (μg(N) m^−3^)	0.35	*0.89*	0.01	0.31	*0.85*	0.17
HNO_3_ + NO_3_^−^ (μg(N) m^−3^)	*0.63*	0.44	0.30	0.11	*0.91*	0.25
% Variance	42.4	29.6	15.8	39.7	25.5	15.7
*PM source*	*LPS*	*Remote sources*	*Residential*/*traffic*	*LPS*	*Remote sources*	*Residential*/*traffic*

Factor loadings >0.6 are represented in italics

As SIA are commonly related with an “external” source of pollution (e.g. Querol et al. 2007; Aarnio et al. 2008), PC2 was identified as remote sources involved in building of background pollution in Poland. PC1 representing by majority of TEs was identified as originating from industrial large point sources (LPS), mainly metallurgy and power generation, concentrated in southern part of the country, as well as close to its southern and eastern boundary, while PC3 with Cr and Cu was attributed to local residential/traffic sources.

TEs are emitted to the atmosphere with fly ash particles created during combustion of solid fuels. Volatile TEs (such as Se and Hg) are completely vaporized at combustion temperatures, while other including As, Pb, Cu, Cd and Zn are partially vaporized at flame temperatures and subsequently condensed on the surfaces of fly ash (Helble 2000; Nelson 2007). Studies of the properties of TEs present in the coal as well as of processes that they undergo during and after coal combustion (e.g. Helble 2000; Senior et al. 2000; Riley et al. 2012) show presence of As, Cd, Co, Pb, Sb and Se in the finest emitted particles—able to be transported over 100 s km—independently of combustion utility and the type of burned coal. This is not, however, a case of Cr and Ni, as their portioning depend on the coal origin and type. Besides, As, Cd, Pb and Zn are typically bonded to the mineral part of coal, while Cr, Cu and Ni are present in both mineral and organic fractions. TEs present in organic parts are more volatile than those bonded with minerals (as As in pyrite), even when coal is burned at low temperatures, typical for domestic stoves. From all these reasons, As, Cd, Pb and Zn can be regarded as markers of regionally transported pollution from LPS, while Cr can be attributed to coal combustion in small-scale installations, as domestic stoves/local boiler houses. Finally, Cu is commonly used as a marker of brake abrasion from road transport (e.g. Schauer et al. 2006; Querol et al. 2007). It can also come from industrial emissions, e.g. Cu, Zn or pyrite smelters (e.g. Bruinen de Bruin et al. 2006); however, the factor loadings of Cu for PC1, characterized by other industrial TEs, were very low (0.04 and 0.30 for 2009 and 2010, respectively). Therefore, both Cr and Cu were considered as originating most likely from local emission sources.

**Fig. 7:**
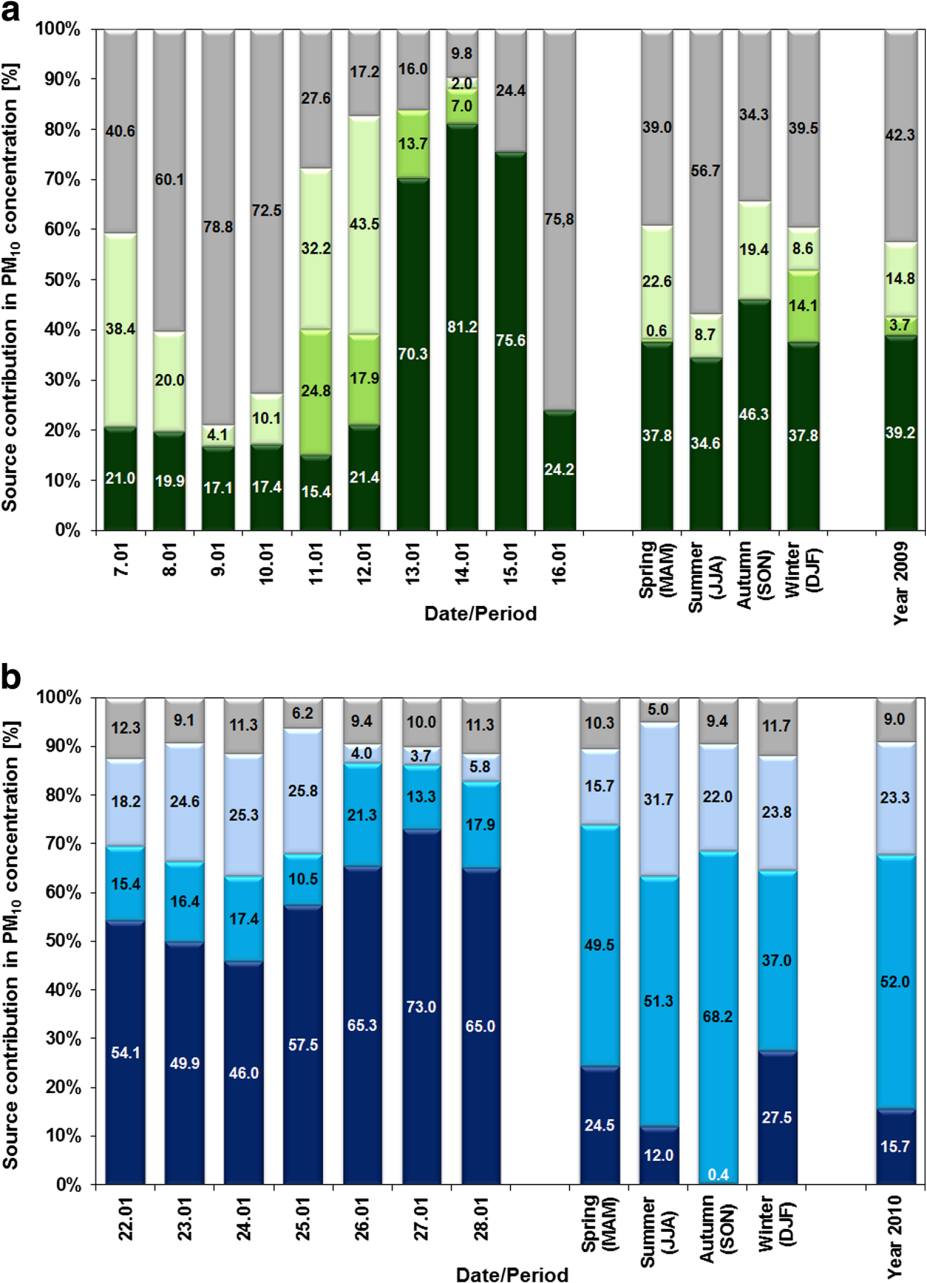
Contributions of PM_10_ emission sources: LPS (*dark colours*), remote (*normal colours*), local pollution (*light colours*), unknown (*grey*), determined in the PCA-MLRA analysis for each day of PM episodes, springs (March–May), summers (June–August), autumns (September–November), winters (December–February) and for the whole years: **a** 2009 and **b** 2010

The contribution of each source type to the PM burden was further quantitatively assessed by means of MLRA. Table 4 evidences the good correspondence between the modelled by PCA-MLRA and the measured PM_10_ concentrations, with *R*^2^ values >0.78 for winter and spring 2010 and *R*^2^ values >0.80 for the rest of seasons. Only for autumn 2009, *R*^2^ value is very low (*R*^2^ = 0.20). Figure 7 presents contributions of identified PM_10_ emission sources for each day of episodes as well as for different seasons and for the whole years 2009 and 2010. During both episodes, LPS had the biggest share in PM_10_ levels during the days with the highest PM concentrations, up to 81 and 73 % in episodes 1 and 2, respectively. They were 2–4 times higher than the whole year averages (16–40 %). In the case of episode 1, the contribution of LPS was around two times higher than the averages for all seasons (35–46 %), while for episode 2, their share was almost three and six times higher than the averages for winter and spring (25–27 %) and for summer (12 %), respectively. The contribution of the remote sources ranged during episode 1 from 7 % to almost 25 %. During episode 2, the share of remote sources (10–21 %) was 2–4 times lower in comparison to winter average (37 %) and to the rest of seasons and the whole year averages (50–68 %). The share of the residential/traffic sources in PM_10_ (2–43 %) at the beginning of the episodes was almost at the same level (episode 2) and 2–3 times lower (episode 1) than the averages for all seasons and the whole years. During the last days of the episodes, five times lower (episode 2) or even no contributions of local sources were noted (episode 1). The share of sources unidentified by PCA-MLRA was relatively small for episode 2 accounting for 6–12 %, while in the early stages of episode 1, the unidentified part was as high as 80 %. Such large share of unidentified sources in episode 1 is surprising; however, it can be expected that it could possibly be related to the various sources which markers are not measured routinely at the Diabla Góra site. According to Chow et al. (2015), the major PM components measured to explain gravimetric mass include (1) anions and cations (2) elements, including metals (up to 51 elements from sodium (Na) to uranium (U)), and (3) OC and EC and their carbon fractions. In Diabla Góra, only a small part of these components are measured; there is a lack of, e.g. chloride (Cl^−^) from anions, water-soluble sodium (Na^+^) and potassium (K^+^), from cations, as well as carbon species. From 51 elements, only 7 were available. Measurement campaign performed at this station in the 2010 shown that in winter, SIA (44 %) and carbon compounds (organic matter, 28 %; EC, 16 %) and Na^+^ with Cl^−^ (11 %) were the main constituents of PM_2.5_ (Rogula-Kozłowska et al. 2014). It could be therefore supposed that contaminated mineral dust, resuspended dust from unpaved roads as well as sea salt are among unidentified sources.

**Table 4:** Correlation between measured PM_10_ concentration (x) and PCA-MLRA results (y) in different seasons of the 2009 and 2010 years

	Slope	*R* ^2^	Intercept
January–March 2009	y = 0.87x	0.90	5.45
April–June 2009	y = 0.88x	0.89	3.19
July–September 2009	y = 0.22x	0.20	10.11
October–December 2009	y = 0.95x	0.95	5.20
January–March 2010	y = 0.82x	0.79	5.55
April–June 2010	y = 0.78x	0.78	5.07
July–September 2010	y = 0.82x	0.82	3.30
October–December 2010	y = 0.85x	0.86	10.09

## Summary and conclusions

The combination of two source apportionment techniques: principal component analysis with multivariate linear regression analysis (PCA-MLRA) and the so-called Lenschow approach, complemented by the analysis of the synoptic situation and variability of the local meteorological parameters as well as of the air mass back trajectories was applied for identification of emission sources determining high PM_10_ concentrations during two episodes of Januaries 2009 and 2010 in Polish urban areas. During the period of 2009–2010, both urban and regional background sites show a statistically significant increase of average PM_10_ concentrations during the heating season (January–March and October–December), presumably due to anthropogenic emissions of primary PM and precursors of secondary PM. The results demonstrate that a number of anthropogenic emission processes and unfavourable synoptic- and local-scale meteorological conditions play an important role in the determination of severe wintertime episodes. It was shown that the episode 2, occurring across the whole country, was associated with the presence of the strong Siberian High ridge over Poland. One of the main findings concerning meteorological influences on PM levels is that depending on the city, the peak PM concentrations occur 2–3 days after the maximum atmospheric pressure and minimum air temperature observed, which are related to the strongest expression of the high-pressure system impact. Three sources of PM_10_ events were identified: remote sources, LPS and residential/traffic sources. At the rural background site, representative for continental background pollution, PM_10_ levels were dominated by LPS situated south-east from it (up to 70–80 % on the days with the highest air pollution), followed by remote and local sources. The Lenschow approach indicate that in central Poland, ambient air pollution was predominantly influenced by LPS, while in the southern cities local residential/traffic sources dominate during the episodes. Although limited number of elements were available in PM, results from analyses suggest that different trace elements characterize coal combustion in large industrial (As, Cd, Pb and Zn) and in small-scale domestic (Cr) furnaces. This study shows that if only routine measurements (as in EMEP network) are available, identification of main PM sources and apportion PM to those sources is possible by the proposed method; however, to provide efficient air quality management tool, more data and more advanced methods are to be used.
